# Supplementary material for: A Systematic, Unbiased Mapping of CD8+ and CD4+ T Cell Epitopes in Yellow Fever Vaccinees
Source: Front Immunol. 2020 Aug 31;11:1836. doi: 10.3389/fimmu.2020.01836 (PMC7489334; doi:10.3389/fimmu.2020.01836)
Supplement: Supplementary file 1 [file Presentation_1.pdf]

Supplementary discussion of the possible bias introduced by the use of an ICS assay to screen for T cell responses.

Prior to the ICS assay, the T cells were expanded by *in vitro* culture with a pool of peptides for 8 days in a media supplemented with IL-2 and IL-15. This could potentially introduce a bias in the epitope discovery process since any T cell reactivity that was not maintained under these conditions could have be missed. A priori, we believe that the epitopes discovered here represents the *ex vivo* anti-virus response since it has been demonstrated, at least for $CD8^+$ T cells, that acute and transient viral infections generate a stable TcR repertoire (Welsh, PubMed Identifier 9841914). This suggests that the specificities of YFV-specific acute and memory T cell responses are identical. Furthermore, had important responses been missed, one would expect that a strictly reverse immunology approach as the one shown in Supplementary Table III would have identified epitopes missed by the HFRI approach. However, when we compared the two approaches and *ex vivo* tested 34 tetramers based on predicted, but not HFRI, identified peptides, we only identified one of these as an epitope missed by the HFRI approach. Thus, we conclude that we have not systematically missed major epitopes.

Supplementary results and discussion of the performance of the hybrid forward-reverse immunology (HFRI) vs reverse immunology approaches.

Additional opportunities arose for comparing the HFRI and reverse immunology approaches. For donor YF1067, the 27 stimulatory 15mer peptides were submitted to the contemporary NetMHCpan 2.4, which returned 156 submer-HLA-I predictions per stimulatory peptide. For each stimulatory peptide, we retrospectively asked how each of the 19 validated epitopes of donor YF1067 ranked amongst the 156 submers predictions. In 23(≈85%) of the 27 submissions, the number one ranking prediction ended up being validated as an epitope (**Table I**). If one applied the contemporary NetMHCpan 2.4 predictor at a stringent %Rank cut-off of 0.5%, it would have correctly identified 17 (89%) of the 19 epitopes (true positives); missed 2 (false negatives), and erroneously included 14 (false positives) leading to a false positive rate of 14/(14+19) = 42%. The current version, NetMHCpan4.0, could have been applied at an even more stringent %Rank cut-off of 0.25%, which would have correctly identified 18 (95%) of the 19 epitopes (true positives); 1 would have been missed (false negative), and 6 would have been erroneously included (false positives) leading to a false positive rate of 6/(6+19) = 24%.

Had the predictors been applied, not to the 27 stimulatory peptides, but to the entire YFV proteome, with the above cut-offs and the HLA-A and -B allotypes of YF1067, the number of false positive predictions included by NetMHCpan 2.4 would have been 187, whereas the current NetMHCpan4.0 would have included 158 false positive predictions.

This supports the general conclusion that the present HFRI approach ranks epitope at the very top of the list of candidates while decimating the false discovery rate. It also demonstrates that the current predictors have improved compared to older versions maintaining a high sensitivity while reducing the false positive rate. Nonetheless, there is still room for further improvements.

A

	C1	C2	C3	C4	C5	C6	C7	C8	C9	C10	C11	C12	C13	C14	C15	C16	C17	C18	C19	C20	C21	C22	C23	C24	C25	C26	C27	C28	C29	C30
R1	25220	25221	25222	25223	25224	[illegible]	25226	25227	25228	25229	25230	25231	25232	25233	25234	25235	25236	25237	25238	25239	25240	25241	25242	25243	25244		25245		25248	
R2	25249	25250	25251	25252	25253	25254	25255	25256	25257	25258	25259	25260	25261	25262	25263	25264	25265	25266	25267	25268	25269	25270	25271	25272	25273	25274	25275	25276	25277	27463
R3	25278	25279	25280	25281	25282	25283	25285	25286	25288	25289	25290	25291	25292	25293	25295	25296	25297	25298	25299	25300	25301	25302	25303	25304	25305	25306	25307	25308	25309	27464
R4	25310	25311	25312	25313	25314	25315	25316	25317	25318	25319	25320	25321	25322	25323	25324	25325	25326	25327	25328	25329	25330	25331	25332	25333	25334	25335	25336	25337	25338	27465
R5	[illegible]	25340	25341	25342	25343	25344	25345	25346	25347	25348	25349	25350	25351	25352	25353	25354	25355	25356	25357	25358	25359	25360	25361	25362	25363	25364	25365	25366		27466
R6	25367	25368	25369	25370	25371	25372	25373	25374	25375	25376	25377	25378	25379	25380	25381	25382	25383	25384	25385	25386	25387	25388	25389	25390	25391	25392	25393	25394	25395	
R7	25396	25397	25398	25399	25400	25401	25402	25406	25407	25412	25413	25414	25415	25416	25417	25418	25419	25420	25421	25422	25423	25425	25426	25427	25428	25429	25430	25431	25432	
R8	25433	25434	25435	25436	25437	25438	25439	25440	25441	25442	25443	25444	25445	25446	25447	25448	25449	25450	25451	25452	25453	25456	25457	25458	25459	25460	25461	25462	25463	27468
R9	25464	25465	25466	25467	25468	25469	25470	25471	25472	25473	25474	25475	25476	25477	25478	25479	25480	25481	25482	25483	25484	25485	25486	25487	25488	25489		25490	25491	
R10	25492	25493	25494	25495	25496	25497	25498	25499	25500	25501	25502	25503	25505	25506	25507	25508	25509	25512	25513	25514	25515	25516	25517	25518	25519	25520	25521	25522	25523	
R11	25524	25525	25526	25527	25528	25529	25530	25531	25532	25533	25534	25536	25537	25538	25539	25540	25541	25542		25545	25546	25547	25548	25549	25550	25551	25552	25553	25554	
R12	25555	25556	25557	25558	25559	25560	25562	25563	25564	25565	25566	25568	25569	25570	25571	25572	25573	25574	25575	25576	25577	25578	25579	25580	25581	25582	25583	25584	25585	27475
R13	25586	25587		25588	25589	25590	25591	25592	25593	25594	25595	25596	25597	25598	25599	25600	25601	25602	25603	25604	25605	25606	25607	25608	25609	25610	25611	25612	25613	27473
R14	25614	25615	25616	25617	25618	25619	25620	25621	25622	25623	25624	25625	25626	25627	25628	25629	25630	25631	25632	25633	25634	25635	25636	25637	25638	25639	25640	25641	25642	
R15	25643	25644	25645	25646	25647	25648	25649	25650	25651	25652	25653	25654	25655	25656	25657	25658	25659	25660	25661	25662	25663	25664	25665	25666	25667	25668	25669	25670	25671	
R16	25672	25673	25674	25675	25676	25677	25678	25679	25680	25681	25682	25683	25684	25685	25686	25687	25688	25689	25690	25691	25692	25693	25694	25695	25696	25697	25698	25699	25700	
R17	25701	25702	25703	25704	25705	25706	25707	25708	25709	25710	25711	25712	25713	25714	25715	25716	25717	25718	25719	25720	25721	25722	25723	25724	25725	25726	25727	25728	25729	
R18	25730	25731	25732	25733	25734	25735	25736	25737	25738	25739	25740	25741	25742	25743	25744	25745	25746	25747	25748	25749	25750	25751	25752		25753	25754	25755	25756	25757	
R19		25762	25763	25764	25765	25766	25767	25768	25769	25770	25771	25772			25775	25776	25777	25778	25779	25782	25783	25784	25785	25786	25787	25788	25789	25790	25791	
R20	25792	25793	25794	25795	25796	25797	25798	25799	25801	25802	25803	25804	25805	25806		25808	25810	25811	25812	25813	25814	25815	25816	25817	25818	25819	25820	25821	25822	27476
R21	25823	25824	25825	25829	25830	25831	25832	25833	25834	25835	25837	25838	25839	25840	25841		25842	25843	25844	25845	25846	25847	25848	25849	25850	25851	25852	25853	25854	27477
R22	25855	25856	25857	25858	25859	25860	25861	25862	25863	25864	25865	25866	25867	25868	25869	25870	25871	25872	25873	25874	25875	25876	25877	25878	25879	25880	25881	25882	25883	
R23	25885	25886	25887	25888	25889	25890	25891	25892	25893	25894	25895	25896	25897	25898	25899	25900	25901	25902	25903	25904	25905	25906	25907	25908	25909	25910	25911	25912	25913	27479
R24	25914	25915	25916	25917	25918	25919	25920	25921	25922	25923	25924	25925	25926	25927	25928	25929	25930	25931	25932	25933	25934	25935	25936	25937	25938	25939	25940	25941	25942	27480
R25	25943	25944	25945	25946	25947	25948	25949	25950	25951	25952	25953	25954	25955	25956	25957	25958	25959	25960	25961	25962	25963	25964	25965	25966	25967	25968	25969	25970	25971	
R26	25972	25973	25974	25975	25976	25977	25978	25979	25980	25981	25982	25983	25984	25985	25986	25987	25988	25989	25990	25991	25992	25993	25994	25995	25996	25997	25998	25999	26000	27471
R27	26001	26002	26003	26004	26005	26006	26007	26008	26009	26010	26011	26012	26013	26014	26015	26016	26017	26018	26019	26020	26021	26022	26023	26024	26025	26026	26027	26028	26029	
R28	26030	26031	26032	26033	26035	26036	26037	26038	26039	26040	26041	26042	26043	26044	26045	26046	26047	26048	26049	26050	26051	26052	26053	26054	26055	26056	26057	26058	26059	
R29	26060	26061	26062	26063	26064	26065	26066	26067	26068	26069	26482	26483	26484	26486	26487	26488	26489	26490	26491	26492	26493	26494	26495	26496	26497	26498	26499	26500	26501	
R30	26502	26503	26504	26505	26506	26507	26508	26509	26510	26511	26512	26513	26514	26515	26516	26517	26518	26520	26521	26522	26523	26524	26525	26526	26527	26528	26529	26530	26531	

B

	1-C1	1-C2	1-C3	1-C4	1-C5	1-C6	1-C7	1-C8	1-C9	1-C10	1-C11	1-C12	1-C13	1-C14	1-C15
1-R1	25310	25311	25312	25313	25314	25315	25316	25317	25318	25319	25320	25321	25322	25323	25324
1-R2	25325	25326	25327	25328	25329	25330	25331	25332	25333	25334	25335	25336	25337	25338	27465
1-R3	25339	25340	25341	25342	25343	25344	25345	25346	25347	25348	25349	25350	25351	25352	25353
1-R4	25354	25355	25356	25357	25358	25359		25360	25361	25362	25363	25364	25365	25366	27466
1-R5	25396	25397	25398	25399	25400	25401	25402	25406	25407	25412	25413	25414	25415	25416	25417
1-R6	25418	25419		25420	25421	25422	25423	25425	25426	25427	25428	25429	25430	25431	25432
1-R7	25433	25434	25435	25436	25437	25438	25439	25440	25441	25442	25443	25444	25445	25446	25447
1-R8	25448	25449	25450	25451	25452	25453	25456	25457	25458	25459	25460	25461	25462	25463	27468
1-R9	25855	25856	25857	25858	25859	25860	25861	25862	25863	25864	25865	25866	25867	25868	25869
1-R10	25870	25871	25872	25873	25874	25875	25876	25877	25878	25879	25880	25881	25882	25883	25715
1-R11	25885	25886	25887	25888	25889	25890	25891	25892	25893	25894	25895	25896	25897	25898	25899
1-R12	25900	25901	25902	25903	25904	25905	25906	25907	25908	25909	25910	25911	25912	25913	26516
1-R13	26001		26002	26003	26004	26005	26006	26007	26008	26009	26010	26011	26012	26013	26014
1-R14	26016	26017	26018	26019	26020	26021	26022	26023	26024	26025		26026	26027	26028	26029
1-R15	25787	25788	25789	25790	25791		25848	25849	25850	25851	25852		26015	27479	

	2-C1	2-C2	2-C3	2-C4	2-C5	2-C6	2-C7	2-C8	2-C9	2-C10	2-C11	2-C12	2-C13	2-C14	2-C15
2-R1	25220	25221	25222	25223	25224	25225	25226	25227	25228	25229	25230	25231	25232	25233	25234
2-R2	25235	25236	25237	25238	25239	25240	25241	25242	25243	25244	25245	25248		27464	
2-R3	25278	25279	25280	25281	25282	25283	25285	25286	25288	25289	25290	25291	25292	25293	25295
2-R4	25296	25297		25298	25299	25300	25301	25302	25303	25304	25305	25306	25307	25308	25309
2-R5	25249	25250	25251	25252	25253	25254	25255	25256	25257	25258	25259	25260	25261	25262	25263
2-R6	25264	25265	25266	25267	25268		25269	25270	25271	25272	25273	25274	25275	25276	25277
2-R7	25367	25368	25369	25370	25371	25372	25373	25374	25375	25376	25377	25378	25379	25380	25381
2-R8	25382	25383	25384	25385	25386	25387	25388	25389	25390	25391	25392	25393	25394	25395	
2-R9	25464	25465	25466	25467	25468	25469	25470	25471	25472	25473	25474	25475	25476	25477	25478
2-R10	25479	25480	25481	25482	25483	25484	25485		25486	25487	25488	25489	25490	25491	27463
2-R11	25555	25556	25557	25558	25559	25560	25562	25563	25564	25565	25566	25568	25569	25570	25571
2-R12	25572	25573	25574	25575	25576	25577	25578	25579	25580	25581	25582	25583	25584	25585	
2-R13	25778	25779	25782	25783	25784	25785	25786	25841	25842	25843	25844	25845	25846	27475	25686
2-R14	25730	25731	25732	25733	25734	25735	25736	25737	25738	25739	25740	25741	25742	25743	25744
2-R15	25745	25746	25747	25748	25749	25750	25751	25752	27477	25753	25754	25755	25756	25757	25847

	3-C1	3-C2	3-C3	3-C4	3-C5	3-C6	3-C7	3-C8	3-C9	3-C10	3-C11	3-C12	3-C13	3-C14	3-C15
3-R1	25492	25493	25494	25495	25496	25497	25498	25499	25500	25501	25502	25503	25505	25506	25507
3-R2	25508	25509	25512	25513	25514	25515	25516	25517	25518	25519	25520	25521	25522	25523	25539
3-R3	25914	25915	25916	25917	25918	25919	25920	25921	25922	25923	25924	25925	25926	25927	25928
3-R4	25929	25930	25931	25932	25933	25934	25935	25936	25937	25938	25939	25940	25941	25942	27480
3-R5	25943	25944	25945	25946	25947	25948	25949	25950	25951	25952	25953	25954	25955	25956	25957
3-R6	25958	25959	25960	25961	25962	25963	25964	25965	25966	25967	25968	25969	25970	25971	25854
3-R7	25972	25973	25974	25975	25976	25977	25978	25979	25980	25981	25982	25983	25984	25985	25986
3-R8	25987	25988	25989	25990	25991	25992	25993	25994	25995	25996	25997	25998	25999	26000	27471
3-R9	26030	26031	26032	26033	26035	26036	26037	26038	26039	26040	26041	26042	26043	26044	26045
3-R10	26046	26047	26048	26049	26050	26051	26052	26053	26054	26055	26056	26057	26058	26059	
3-R11	26060	26061	26062	26063	26064	26065	26066	26067	26068	26069	26482	26483	26484	26486	26487
3-R12	26488	26489	26490	26491	26492	26493	26494	26495	26496	26497	26498	26499	26500	26501	27473
3-R13	25792	25793	25794	25795	25796	25797	25798	25799	25801	25802	25803	25804	25805	25806	25628
3-R14	25808	25810	25811	25812	25813	25814	25815	25816	25817	25818	25819	25820	25821	25822	27476
3-R15	25769	25770	25771	25772	25775	25776	25777	25833	25834	25835	25837	25838	25839	25840	25657

	4-C1	4-C2	4-C3	4-C4	4-C5	4-C6	4-C7	4-C8	4-C9	4-C10	4-C11	4-C12	4-C13	4-C14
4-R1	25524	25525	25526	25527	25528	25529	25530	25531	25532	25533	25534	25536	25537	25538
4-R2	25540	25541	25542	25545	25546	25547	25548	25549	25550	25551	25552	25553	25554	25853
4-R3	25586	25587	25588	25589	25590	25591	25592	25593	25594	25595	25596	25597	25598	25599
4-R4	25600	25601	25602	25603	25604	25605	25606	25607	25608	25609	25610	25611	25612	25613
4-R5	25614	25615	25616	25617	25618	25619	25620	25621	25622	25623	25624	25625	25626	25627
4-R6	25629	25630	25631	25632	25633	25634	25635	25636	25637	25638	25639	25640	25641	25642
4-R7	25643	25644	25645	25646	25647	25648	25649	25650	25651	25652	25653	25654	25655	25656
4-R8	25658	25659	25660	25661	25662	25663	25664	25665	25666	25667	25668	25669	25670	25671
4-R9	25672	25673	25674	25675	25676	25677	25678	25679	25680	25681	25682	25683	25684	25685
4-R10	25687	25688	25689	25690	25691	25692	25693	25694	25695	25696	25697	25698	25699	25700
4-R11	25701	25702	25703	25704	25705	25706	25707	25708	25709	25710	25711	25712	25713	25714
4-R12	25716	25717	25718	25719	25720	25721	25722	25723	25724	25725	25726	25727	25728	25729
4-R13	26502	26503	26504	26505	26506	26507	26508	26509	26510	26511	26512	26513	26514	26515
4-R14	26517	26518	26520	26521	26522	26523	26524	26525	26526	26527	26528	26529	26530	26531
4-R15	25762	25763	25764	25765	25766	25767	25768	25823	25824	25825	25829	25830	25831	25832

Supplementary Figure S1. Using peptide matrices to screeen for peptides containing YFV-derived T cell epitopes
A) The 30x30 matrix approach. The 870 Yellow Fever peptides were organized into a 30x30 matrix. Each 5 digit number in the matrix figure signifies a specific peptide. Row and column peptide pools were made: row pools by mixing the ca. 30 different peptides listed horizontally next to the row number (R1-R30) giving in total 30 different row pools; column pools by mixing the ca. 30 different peptides listed vertically below the column number (C1-C30) giving in total 30 different column pools. This means that every single peptide is present in one specific column and one specific row pool (the oval exemplifies one peptide, 25344, which was present in both column 6 and row 5). The 60 peptide pools (30 row pools and 30 column pools) were used to stimulate the T cells and responses against each row and column pools were evaluated by ELISpot. Theoretically, any given peptide epitope should give rise to a positive response in both a row and a column pool and should therefore be identified as the intersection between the positive row and column pool. The more epitopes, the more positive row and column pools, the more intersections. As exemplified in green, positive responses in both pool R10, R18, C9, and C22, could be due to T cells recognizing two or more of the four peptides (25500, 25738, 25751, and 25751) “located” in the intersection between these row and column pools. Thus, when evaluating T cell responses every intersection of T cell stimulatory column and row pools tentatively identified a T cell epitope-containing peptide for further deconvolution and study.

B) The 4x(15x15) matrices approach. The 870 Yellow Fever peptides listed in the 30x30 matrix was reorganized into four 15x15 matrices. Following the same general pooling strategy as for the 30x30 matrix, the 4x(15x15) matrix strategy led to the generation of 4x15 column pools and 4x15 row pools (actually, matrix 4 only contained 14 columns). Per submatrix, every intersection of T cell stimulatory column and row pools tentatively identified a T cell epitope-containing peptide for further deconvolution and study.

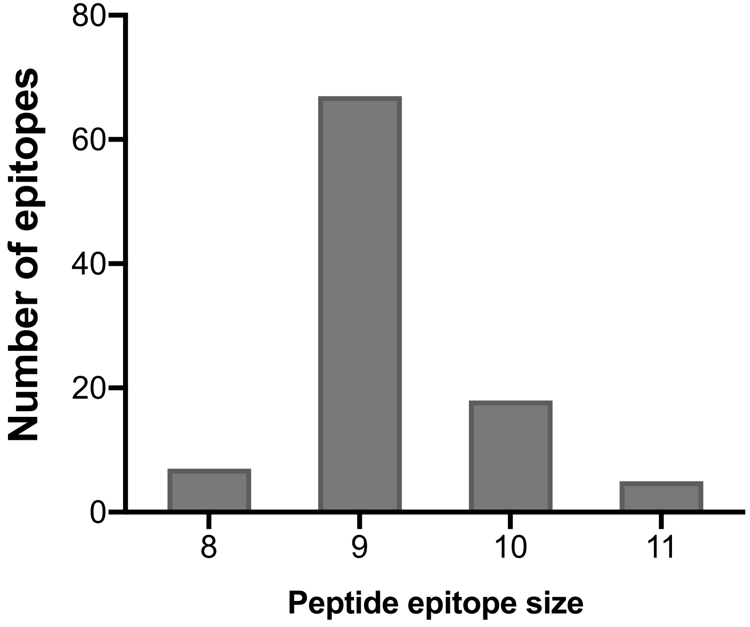

Supplementary Figure S2. The length distribution of the identified CD8+ T cell epitopes.
Shown is the number of identified YFV-specific, HLA-class I restricted CD8+ T cell epitopes of 8-, 9-, 10- and 11-mer size for all tetramer validated epitopes.

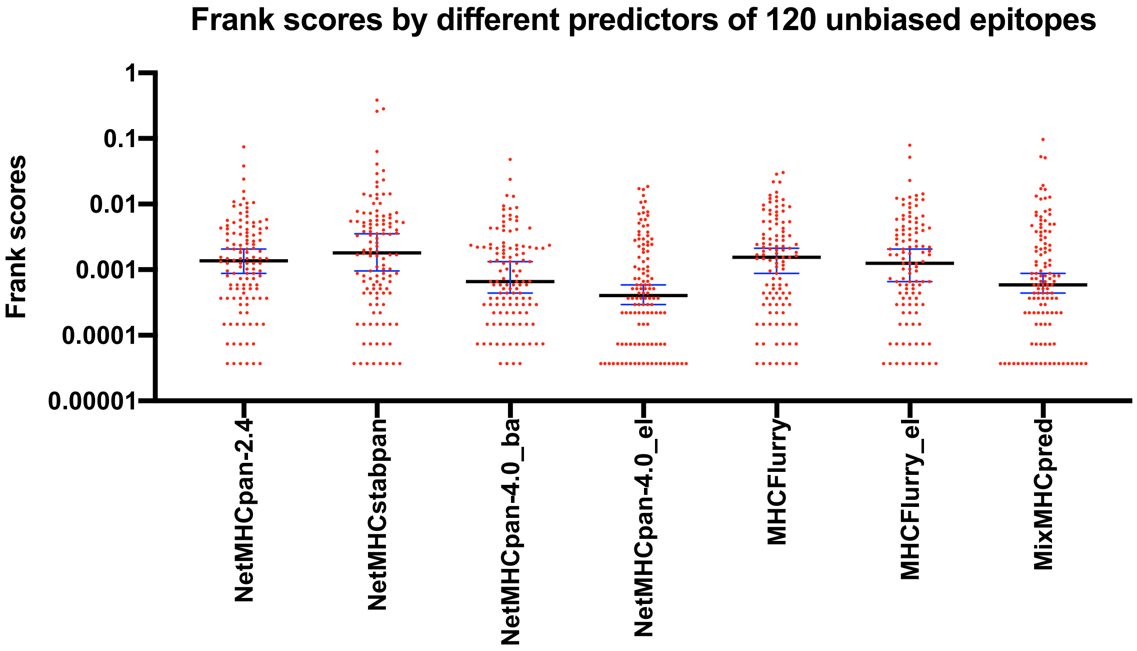

	NetMHCpan-2.4	NetMHCstabpan	NetMHCpan-4.0_ba	NetMHCpan-4.0_el	MHCFlurry	MHCFlurry_el	MixMHCpred
AUC	0.99636	0.98743	0.99755	0.99797	0.99631	0.99604	0.99562
Median Frank score	0.00136	0.00180	0.00066	0.00040	0.00154	0.00125	0.00059
Wilcoxon matched-pairs signed rank test. p-value	0.0001	<0.0001	0.0102	NA	<0.0001	<0.0001	0.0156

Supplementary Figure S3. The Yellow Fever proteome of 3411 aa is submitted to the various indicated predictors along with the HLA-I restriction elements used by the epitopes discovered by the HFRI approach, and the bindings of all 13610 possible 8-11mer peptides per HLA-I are predicted. Per predictor, the median Frank scores (the frequencies of False Positive predictions) of the 120 HFRI discoverted epitopes is determined. The median Frank is indicated by black bars and the +/- 95% CI is indicated by blue error bars. For illstrative purposes, the perfect predictions with a Frank score of 0 has been recoded to a Frank score of 0.000037 and are clearly visible as the bottom line of symbols.

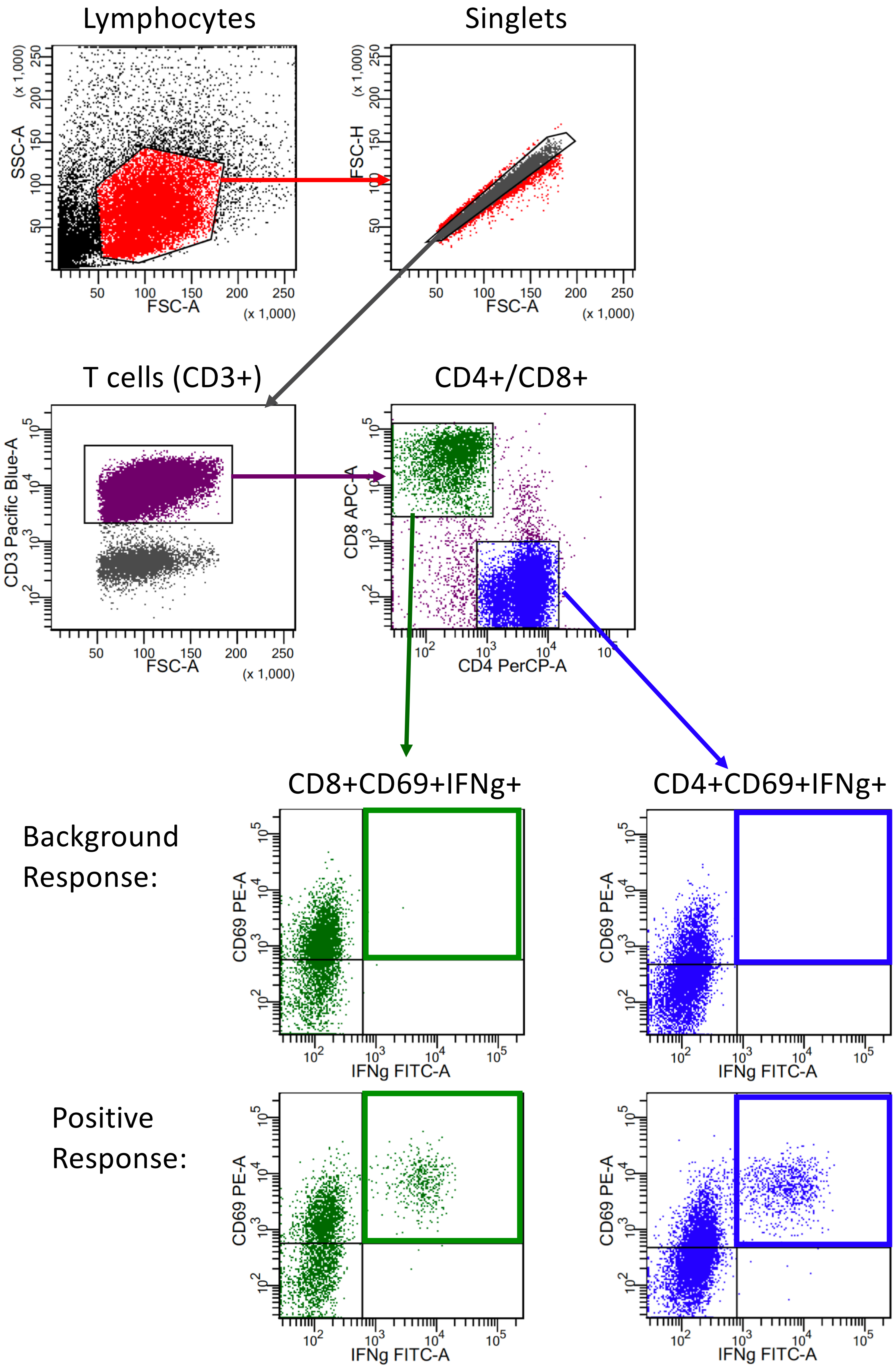

Supplementary Figure S4: Gating strategy for intracellular cytokine staining (ICS). Initially a lymphocyte gate is set based on FSC-A/SSC-A, then the singlets are gated based on FCS-A/FCS-H before the CD3+ T cells are gated. Within the gated CD3+ T cell population the CD4+ and CD8+ T cells are gated. The gated CD4+ and CD8+ T cell are then analysed in a CD69/IFNg plot and the responding T cells CD4+CD69+IFNg+ and CD8+CD69+IFNg+, respectively, can be determined. Illustrated here are background plots (no stimulation) and positive response plots (stimulated with a peptide epitope) for both CD4+CD69+IFNg+ and CD8+CD69+IFNg+.

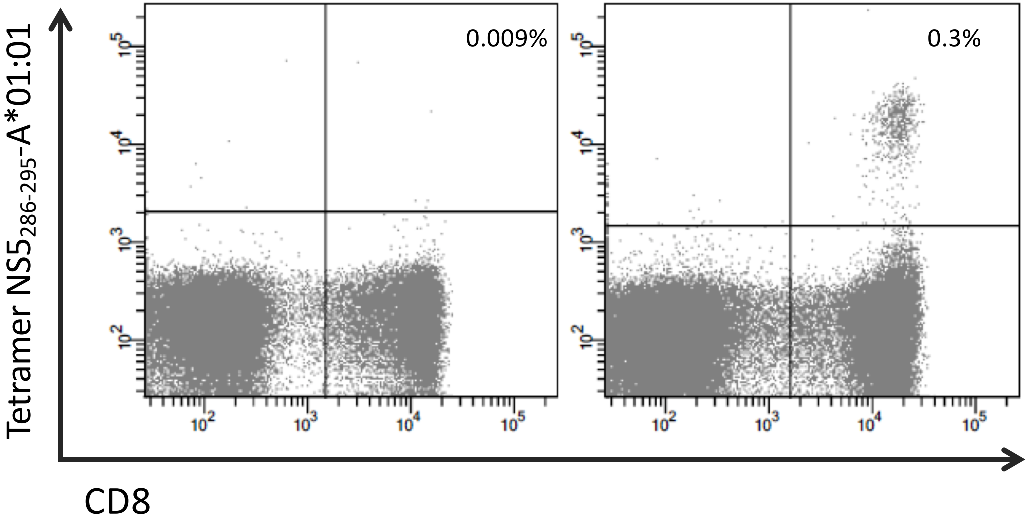

Figure S5: HLA-A*01:01 positive donor stained for the dominant HLA-A1*01:01 epitope, NS5286-295. PBMCs from the donor before vaccination (left plot) and after Yellow Fever vaccination (right plot) were stained with anti CD3-Pacific Blue, CD8-PerCp, and PE labeled NS5286-295-A1*01:01 tetramer. Shown is CD3 gated T cells and the percentage given is tetramer +CD8+ T cells.

Proteom position	Protein position	Sequence	HLA restriction	Predicted affinity (%RANK)	Stability ($t_{1/2}$; h)	Responders /donor tested	Tetramer validated (%$CD8^+$ T cells)	Reference
YFV_pp61-69	CapsidC (61-69)	ITAHLKRLW	B*57:01	0.05	9.0	3/11	0.01-0.02	
YFV_pp64-72	CapsidC (64-72)	HLKRLWKML	B*08:01	0.30	0.5	8/42	0.03-0.30	
YFV_pp102-110	ER anchor (1-9)	SHDVLTVQF	B*38:01	0.40	1.6	7/7	0.02-0.20	
YFV_pp103-110	ER anchor (2-9)	HDVLTVQF	B*37:01	3.00	19.4	2/2	0.02-0.50	
YFV_pp331-339	EnvE (46-54)	ETVAIDRPA	A*68:02	0.18	15.8	2/2	0.10-0.40	
YFV_pp453-460	EnvE (168-175)	QEVEFIGY	B*18:01	0.08	4.1	13/14	0.01-0.70	
YFV_pp471-479	EnvE (186-194)	TAVDFGNSY	B*35:01	0.25	2.7	9/23	0.01-0.10	
YFV_pp485-494	EnvE (200-209)	TESWIVDRQW	B*44:02	0.15	NA	33/35	0.01-1.50	(PMID:28386132)[1]
YFV_pp485-494	EnvE (200-209)	TESWIVDRQW	B*44:03	0.13	NA	8/12	0.03-0.20	
YFV_pp486-494	EnvE (201-209)	ESWIVDRQW	B*57:01	0.30	1.9	13/15	0.01-0.20	
YFV_pp492-500	EnvE (207-215)	RQWAQDLTL	B*40:02	0.40	2.4	2/7	0.01-0.20	
YFV_pp492-500	EnvE (207-215)	RQWAQDLTL	B*13:02	0.40	2.4	4/4	0.05-0.30	(PMID:28386132)[1]
YFV_pp500-510	EnvE (215-225)	LPWQSGSGGVW	B*53:01	0.03	NA	3/3	0.20-0.30	
YFV_pp500-510	EnvE (215-225)	LPWQSGSGGVW	B*35:01	0.20	2.9	12/22	0.01-0.20	
YFV_pp511-519	EnvE (226-234)	REMHHLVEF	B*37:01	0.03	19.8	3/3	0.01-0.30	
YFV_pp511-519	EnvE (226-234)	REMHHLVEF	B*40:01	0.05	1.5	19/30	0.01-0.80	
YFV_pp517-525	EnvE (232-240)	VEFEPPHAA	B*40:02	0.80	12.1	6/7	0.01-0.10	
YFV_pp517-525	EnvE (232-240)	VEFEPPHAA	B*50:01	0.10	3.8	2/3	0.10-0.20	
YFV_pp569-577	EnvE (284-292)	RVKLSALTL	B*07:02	1.50	4.8	5/41	0.03-0.40	(PMID: 22039500)[2]
YFV_pp569-578	EnvE (284-293)	RVKLSALTLK	A*03:01	0.25	36.3	29/39	0.01-0.20	
YFV_pp651-660	EnvE (366-375)	EVNPPFGDSY	A*25:01	0.13	3.1	2/11	0.03-0.10	
YFV_pp651-660	EnvE (366-375)	EVNPPFGDSY	A*26:01	0.13	1.5	3/10	0.02-0.03	
YFV_pp729-737	EnvE (444-452)	GLFGGLNWI	A*02:01	0.30	5.1	12/89	0.01-0.10	(PMID: 22039500)[2]
YFV_pp759-767	EnvE (474-482)	SMSMILVGV	A*02:01	0.30	16.4	27/95	0.01-0.10	
YFV_pp802-810	NS1 (24-32)	DSDDWLNKY	A*01:01	0.05	1.4	40/53	0.01-0.20	
YFV_pp883-891	NS1 (105-113)	SRIRDGLQY	B*27:02	0.80	9.0	1/1	0.05-0.05	
YFV_pp884-893	NS1 (106-115)	RIRDGLQYGW	A*32:01	0.40	107.9	15/18	0.02-0.10	
YFV_pp884-893	NS1 (106-115)	RIRDGLQYGW	B*57:01	0.40	6.3	9/15	0.01-0.10	
YFV_pp885-893	NS1 (107-115)	IRDGLQYGW	B*27:02	1.00	2.4	1/1	0.40-0.40	
YFV_pp894-902	NS1 (116-124)	KTWGKNLVF	A*32:01	0.03	32.1	19/19	0.10-2.80	(PMID:28386132)[1]
YFV_pp894-902	NS1 (116-124)	KTWGKNLVF	B*58:01	0.25	9.3	3/3	0.20-0.40	
YFV_pp894-902	NS1 (116-124)	KTWGKNLVF	B*57:01	0.10	5.3	14/15	0.02-0.80	(PMID:25674793)[1]
YFV_pp945-953	NS1 (167-175)	VYMDAVFEY	A*29:02	0.08	39.1	3/9	0.03-0.10	
YFV_pp945-953	NS1 (167-175)	VYMDAVFEY	A*24:02	0.80	32.8	7/33	0.01-0.20	(PMID 23338234)[3]
YFV_pp970-978	NS1 (192-200)	KSAHGSPTF	B*58:01	0.13	13.7	2/3	0.10-0.10	
YFV_pp983-991	NS1 (205-213)	HEVNGTWMI	B*40:01	0.13	1.2	2/31	0.02-0.03	
YFV_pp1002-1010	NS1 (224-232)	CEWPLTHTI	B*49:01	0.01	11.7	2/3	0.05-0.05	
YFV_pp1002-1010	NS1 (224-232)	CEWPLTHTI	B*40:01	0.40	0.9	6/26	0.02-0.80	
YFV_pp1114-1123	NS1 (336-345)	RPRKTHESHL	B*07:02	0.03	4.8	19/44	0.01-0.20	
YFV_pp1114-1124	NS1 (336-346)	RPRKTHESHLV	B*07:02	0.10	3.0	12/44	0.01-0.20	
YFV_pp1119-1127	NS1 (341-349)	HESHLVRSW	B*44:02	0.05	NA	30/33	0.02-0.40	
YFV_pp1119-1127	NS1 (341-349)	HESHLVRSW	B*44:03	0.05	NA	9/13	0.10-0.30	
YFV_pp1131-1140	NS2A (1-10)	GEIHAVPFGL	B*40:01	0.01	1.5	22/28	0.01-0.50	
YFV_pp1131-1141	NS2A (1-11)	GEIHAVPFGLV	B*40:01	0.25	1.2	13/31	0.01-0.30	
YFV_pp1134-1143	NS2A (4-13)	HAVPFGLVSM	B*35:08	0.80	13.5	2/2	1.30-9.70	(PMID: 11853408)[4]; (PMID:19740333)[5]
YFV_pp1134-1143	NS2A (4-13)	HAVPFGLVSM	B*35:03	0.80	7.7	6/6	0.20-4.50	(PMID: 11853408)[4]; (PMID:19740333)[5]
YFV_pp1134-1143	NS2A (4-13)	HAVPFGLVSM	B*35:01	0.40	7.0	22/24	0.10-1.70	(PMID: 11853408)[4]; (PMID:19740333)[5]; (PMID:28386132)[1]
YFV_pp1200-1207	NS2A (70-77)	DAMYMALI	B*51:01	0.80	17.3	13/18	0.03-2.20	
YFV_pp1227-1234	NS2A (97-104)	SPRERLVL	B*07:02	0.01	4.1	4/44	0.01-0.02	
YFV_pp1227-1236	NS2A (97-106)	SPRERLVLTL	B*07:02	0.03	1.8	40/44	0.02-4.00	
YFV_pp1317-1325	NS2A (187-195)	SMQKTIPLV	A*02:01	0.80	27.3	43/95	0.01-0.10	
YFV_pp1397-1405	NS2B (43-51)	SVAGRVDGL	A*02:05	4.00	18.7	5/5	0.02-0.40	
YFV_pp1464-1472	NS2B (110-118)	HPFALLLVL	B*35:01	0.30	5.9	22/23	0.05-0.90	(PMID: 11853408)[4]; (PMID:19740333)[5]; (PMID:28386132)[1]
YFV_pp1464-1472	NS2B (110-118)	HPFALLLVL	B*35:03	0.13	5.9	5/6	0.20-0.80	(PMID: 11853408)[4]; (PMID:19740333)[5]; (PMID:28386132)[1]
YFV_pp1471-1479	NS2B (117-125)	VLAGWLFHV	A*02:01	0.01	27.7	75/86	0.01-0.50	(PMID: 22039500)[2]
YFV_pp1488-1496	NS3 (4-12)	VLWDIPTPK	A*11:01	0.50	9.0	16/28	0.01-0.20	
YFV_pp1488-1496	NS3 (4-12)	VLWDIPTPK	A*03:01	0.18	6.2	31/40	0.01-0.40	(PMID:28386132)[1]
YFV_pp1508-1516	NS3 (24-32)	IYGIFQSTF	A*24:02	0.08	40.7	22/33	0.01-1.00	(PMID:28386132)[1]
YFV_pp1508-1516	NS3 (24-32)	IYGIFQSTF	A*23:01	0.13	40.6	4/5	0.01-0.20	
YFV_pp1557-1565	NS3 (73-81)	SVKEDLVAY	B*15:01	0.80	14.5	28/30	0.03-1.10	(PMID:28386132)[1]
YFV_pp1557-1565	NS3 (73-81)	SVKEDLVAY	B*35:01	1.50	3.2	13/23	0.01-0.10	(PMID:28386132)[1]
YFV_pp1608-1615	NS3 (124-131)	GEIGAVAL	B*40:01	0.01	3.2	5/17	0.02-0.10	

YFV_pp1632-1641	NS3 (148-157)	EVIGLYGNGI	A*68:02	0.50	2.9	1/2	0.04-0.04	
YFV_pp1635-1643	NS3 (151-159)	GLYGNGILV	A*02:01	1.50	20.1	27/80	0.01-0.20	(PMID: 22039500)[2]
YFV_pp1690-1697	NS3 (206-213)	RRFLPQIL	C*06:02	0.15	1.4	6/28	0.10-2.78	
YFV_pp1689-1697	NS3 (205-213)	TRRFLPQIL	C*06:02	8.00	17.7	27/28	0.02-1.90	
YFV_pp1702-1710	NS3 (218-226)	RRRLRTLVL	B*14:02	0.15	0.9	4/4	0.05-0.40	(PMID: 18762226)[6]
YFV_pp1718-1727	NS3 (234-243)	SEMKEAFHGL	B*40:01	0.05	1.3	16/29	0.01-0.40	
YFV_pp1728-1736	NS3 (244-252)	DVKFHTQAF	A*25:01	0.15	3.2	10/11	0.02-0.30	(PMID: 18762226)[6]
YFV_pp1770-1778	NS3 (286-294)	IIMDEAHFL	A*02:05	0.20	20.7	5/5	0.10-0.70	
YFV_pp1770-1778	NS3 (286-294)	IIMDEAHFL	A*02:01	0.50	9.2	43/93	0.01-0.10	(PMID: 22039500)[2]
YFV_pp1777-1785	NS3 (293-301)	FLDPASIAA	A*02:01	0.40	9.9	56/84	0.01-0.70	
YFV_pp1831-1839	NS3 (347-355)	EPWNTGHDW	B*53:01	0.10	NA	2/3	0.10-0.10	
YFV_pp1910-1918	NS3 (426-434)	RVLDCRTAF	A*32:01	0.80	31.1	4/19	0.05-0.10	
YFV_pp1991-1999	NS3 (507-515)	GMVAPLYGV	A*02:01	0.10	9.4	37/96	0.01-0.20	
YFV_pp2030-2039	NS3 (546-555)	LPVWLSWQVA	B*56:01	0.05	2.3	3/3	0.10-0.10	
YFV_pp2060-2068	NS3 (576-584)	ILNDSGETV	A*02:01	1.50	12.2	42/92	0.01-0.40	
YFV_pp2128-2136	NS4A (21-29)	GEAMDTISV	B*40:01	0.40	1.3	7/32	0.01-0.20	
YFV_pp2129-2137	NS4A (22-30)	EAMDTISVF	B*35:01	0.10	3.7	3/21	0.01-0.03	
YFV_pp2130-2138	NS4A (23-31)	AMDTISVFL	A*02:01	0.80	11.0	5/95	0.01-0.03	(PMID:27017899)
YFV_pp2152-2160	NS4A (45-53)	SMMPEAMTI	B*52:01	0.10	9.1	3/3	0.01-0.03	
YFV_pp2154-2163	NS4A (47-56)	MPEAMTIVML	B*35:03	0.05	3.9	5/6	0.10-0.80	
YFV_pp2154-2163	NS4A (47-56)	MPEAMTIVML	B*35:01	0.30	3.8	21/25	0.03-1.00	
YFV_pp2154-2163	NS4A (47-56)	MPEAMTIVML	B*53:01	0.15	2.9	2/3	0.10-0.10	
YFV_pp2155-2163	NS4A (48-56)	PEAMTIVML	B*40:01	1.00	ND	13/17	0.10-0.80	
YFV_pp2180-2189	NS4A (73-82)	SPKGISRMSM	B*07:02	0.10	8.6	29/43	0.01-0.10	
YFV_pp2389-2397	NS4B (133-141)	KLAQRRVFH	A*03:01	1.00	8.6	26/32	0.01-1.40	
YFV_pp2421-2429	NS4B (165-173)	ALYEKKLAL	A*02:01	2.00	19.2	3/85	0.01-0.50	
YFV_pp2421-2429	NS4B (165-173)	ALYEKKLAL	B*08:01	0.25	0.4	28/41	0.01-0.20	
YFV_pp2423-2431	NS4B (167-175)	YEKKLALYL	B*40:02	0.40	24.4	7/7	0.04-0.60	
YFV_pp2423-2431	NS4B (167-175)	YEKKLALYL	B*40:01	0.30	1.1	4/28	0.03-0.30	
YFV_pp2470-2478	NS4B (214-222)	LLWNGPMAV	A*02:01	0.13	38.2	98/98	0.03-11.50	(PMID 19933869)
YFV_pp2494-2502	NS4B (238-246)	VMYNLWKMK	A*03:01	0.05	0.8	17/38	0.01-2.40	
YFV_pp2524-2533	NS5 (18-27)	LLDKRQFELY	A*01:01	0.05	11.7	32/52	0.01-0.20	
YFV_pp2571-2579	NS5 (65-73)	FHERGYVKL	B*38:01	0.10	5.2	7/7	0.10-0.60	
YFV_pp2571-2579	NS5 (65-73)	FHERGYVKL	B*39:01	0.15	0.6	5/6	0.10-1.00	
YFV_pp2669-2677	NS5 (163-171)	RVLDTVEKW	A*32:01	0.80	101.6	11/19	0.01-0.10	
YFV_pp2669-2677	NS5 (163-171)	RVLDTVEKW	B*58:01	0.13	32.9	2/3	0.10-0.40	
YFV_pp2669-2677	NS5 (163-171)	RVLDTVEKW	B*57:01	0.15	16.2	13/15	0.01-0.20	
YFV_pp2706-2714	NS5 (200-208)	RRFGGTVIR	B*27:05	0.05	22.9	6/12	0.02-0.10	
YFV_pp2711-2719	NS5 (205-213)	TVIRNPLSR	A*68:01	0.50	11.9	11/19	0.03-0.70	
YFV_pp2723-2731	NS5 (217-225)	HEMYYVSGA	B*50:01	0.08	NA	3/3	0.04-0.10	
YFV_pp2762-2770	NS5 (256-264)	DVILPIGTR	A*68:01	0.50	37.4	20/20	0.01-0.90	
YFV_pp2792-2801	NS5 (286-295)	KSEYMTSWFY	A*01:01	0.08	44.2	50/52	0.01-4.60	(PMID:28386132)[1]
YFV_pp2854-2862	NS5 (348-356)	TPFGQQRVF	B*35:01	0.40	3.6	8/22	0.01-0.10	
YFV_pp2879-2887	NS5 (373-381)	KIMKVVNRW	B*58:01	0.18	19.2	1/3	0.01-0.01	
YFV_pp2882-2890	NS5 (376-384)	KVVNRWLFR	A*03:01	0.40	6.5	2/38	0.02-0.03	
YFV_pp2974-2982	NS5 (468-476)	KAKGSRAIW	B*57:01	0.13	11.2	10/15	0.01-0.10	
YFV_pp2977-2985	NS5 (471-479)	GSRAIWYMW	B*57:01	0.08	9.1	5/15	0.01-0.02	
YFV_pp2981-2990	NS5 (475-484)	IWYMWLGARY	A*29:02	0.10	0.6	2/8	0.10-0.20	
YFV_pp2982-2990	NS5 (476-484)	WYMWLGARY	A*29:02	0.25	24.7	8/9	0.10-0.50	(PMID:28386132)[1]
YFV_pp2983-2990	NS5 (477-484)	YMWLGARY	A*29:02	0.03	3.0	3/9	0.03-0.30	
YFV_pp2983-2990	NS5 (477-484)	YMWLGARY	A*01:01	1.50	67.3	47/52	0.01-0.80	
YFV_pp3024-3032	NS5 (518-526)	YVIRDLAAM	B*35:01	0.13	5.9	16/20	0.01-0.10	
YFV_pp3063-3071	NS5 (557-565)	YMSPHHKKL	A*02:05	1.50	16.9	4/5	0.10-0.20	
YFV_pp3178-3186	NS5 (672-680)	RPIDDRFGL	B*07:02	0.15	6.3	45/45	0.01-0.80	(PMID: 23338234)[3]; (PMID: 28386132)[1]
YFV_pp3178-3187	NS5 (672-681)	RPIDDRFGLA	B*07:02	0.80	3.1	8/46	0.01-0.50	
YFV_pp3178-3188	NS5 (672-682)	RPIDDRFGLAL	B*07:02	0.03	2.5	41/47	0.01-0.60	
YFV_pp3178-3188	NS5 (672-682)	RPIDDRFGLAL	B*35:03	0.25	3.5	3/6	0.01-0.10	
YFV_pp3389-3399	NS5 (883-893)	YTDYLTVMDRY	A*01:01	0.01	42.0	48/53	0.06-0.70	

Table SI. A complete list of the 120 peptide-specific, HLA-I-restricted CD8+ T cell epitopes identified by the HFRI approach.

The HFRI-based $CD8^+$ T cell epitope discovery process detailed for donor YF1067 was extended to 50 randomely selected, primary YFV-vaccinated donors. Ninety-two different epitopes restricted by 40 HLA-I allotypes were discovered. The peptide-HLA-I affinity was predicted by NetMHCpan 2.4 (given in %Rank; the lower, the better); the stability was measured (given as half-life at 37°C in hours; the longer, the better). Productively interaction peptide-HLA-I combinations were used to design peptide-HLA-I tetramers. the resulting tetramers were used to stain and ex vivo analyze CD8+ T cells by flow cytometry gating on CD3+ CD8+ T cells. The prevalence of these responses is given as "the number of positive donors/the number of donors tested". The magnitudes are given as the range of frequencies (in %) of *ex vivo* tetramer-stained CD8+ T cells. Note, that for the prevalence and magnitude measurements, the specific responses identified in 50 vaccinees was extended to additional donors within our cohort of primary vaccinee, who expressed the relevant HLA-I allotype.
PubMed ID (PMID) references are given in the event that an epitope had been reported before this study.

Proteome position	Protein position	Sequence	HLA restriction	Predicted affinity (%RANK)	Stability ($t_{½}$; h)	Responders per donors tested	Tetramer validated (%CD8^{+} T cells)
YFV_pp438-446	EnvE (153-161)	NTDIKTLKF	A*01:01	0.15	1.70	46/51	0.02-0.90
YFV_pp511-519	EnvE (226-234)	REMHHLVEF	B*40:02	0.03	17.7	3/7	0.01-0.02
YFV_pp1131-1140	NS2A (1-10)	GEIHAVPFGL	B*40:02	0.05	11.8	5/7	0.03-0.10
YFV_pp1504-1512	NS3 (20-28)	LEDGIYGIF	B*40:01	0.4	2.30	6/27	0.01-0.10
YFV_pp2762-2770	NS5 (256-264)	DVILPIGTR	A*33:01	0.18	NA	2/2	0.01-0.10
YFV_pp3024-3032	NS5 (518-526)	YVIRDLAAM	A*26:01	0.01	10.6	7/10	0.01-0.30
YFV_pp3089-3098	NS5 (583-592)	RPAPGGKAYM	B*07:02	0.03	8.5	22/44	0.01-0.20
YFV_pp3116-3124	NS5 (610-618)	ALNTITNLK	A*03:01	0.10	14.8	12/33	0.01-0.10
YFV_pp3268-3276	NS5 (762-770)	YANMWSLMY	B*35:01	0.30	8.6	7/22	0.01-0.10

Supplementary Table SII. CD8+ T cell epitopes derived from past epitope discovery efforts.

A panel of 533 YFV-derived peptides obtained from past epitope discovery efforts were evaluated by NetMHCpan 2.4 in an effort to discover additional CD8+ T cell epitopes. The stability of predicted peptide-HLA-I binders were verified experimentally. An additional 90 peptide-HLA-I tetramers could be generated and tested in appropriate primary YFV-vaccinated donors. Additionally 9 epitopes and their restriction elements were identified. The prevalence and response magnitude of the corresponding CD8+ T cell responses were evaluated as descibed in Supplementary Table SI.

		Predicted	Measured	T cell analysis	
**Protein Position**	**Sequence**	**%Rank**	**Stability (T½, h)**	**Responders /donor tested**	**Suggested by HFRI**
NS2A (97-104)	SPRERLVL	0.01	4.1	4/44	Yes[1]
NS1 (243-251)	MPRSIGGPV	0.03	4.6	0/18	
NS2A (97-106)	SPRERLVLTL	0.03	1.8	40/44	Yes[1]
NS5 (672-682)	RPIDDRFGLAL	0.03	2.5	41/47	Yes
NS1 (336-345)	RPRKTHESHL	0.03	4.8	19/44	Yes
NS5 (583-592)	RPAPGGKAYM	0.03	8.5	22/44	No
NS4B (163-173)	MPALYEKKLAL	0.05	2.0	0/16	
CapsidC (15-23)	MVRRGVRSL	0.05	3.3	0/16	
NS5 (112-119)	KPMNVQSL	0.05	3.4	0/18	
NS5 (246-253)	RPTGKVTL	0.08	6.5	0/18	
NS1 (336-346)	RPRKTHESHLV	0.10	3.0	12/44	Yes
NS4A (73-82)	SPKGISRMSM	0.10	8.6	29/43	Yes[2]
NS4A (47-55)	MPEAMTIVM	0.10	0.8	0/17	Yes[3]
NS4B (218-228)	GPMAVSMTGVM	0.10	14.4	0/16	
NS3 (133-142)	YPSGTSGSPI	0.10	2.5	0/17	
prM (128-136)	NPFFAVTAL	0.12	2.3	0/18	
NS3 (367-376)	LPSIRAANVM	0.12	5.7	0/18	
NS4B (107-117)	MPLLCGIGCAM	0.12	3.5	0/18	
NS1 (102-111)	HPFSRIRDGL	0.12	3.0	0/16	
NS5 (672-680)	RPIDDRFGL	0.15	6.3	45/45	Yes
NS5 (559-569)	SPHHKKLAQAV	0.15	1.4	0/18	
NS5 (259-266)	LPIGTRSV	0.17	0.7	0/18	Yes
NS3 (199-209)	HPGAGKTRRFL	0.20	1.6	0/17	
NS2B (110-118)	HPFALLLVL	0.20	NF	ND	
NS5 (710-720)	VPFCSHHFHEL	0.25	3.9	0/18	
NS4A (100-108)	KPTHISYVM	0.25	6.5	0/18	
NS1 (266-273)	GPWMQVPL	0.25	0.4	0/18	
NS5 (185-192)	APYMPDVL	0.25	6.4	0/18	
NS3 (361-370)	RPTAWFLPSI	0.25	1.7	0/17	
NS1 (226-236)	WPLTHTIGTSV	0.25	2.6	0/18	
NS5 (583-590)	RPAPGGKA	0.25	NA	ND	
EnvE (52-59)	RPAEVRKV	0.25	NA	ND	
NS4A (47-56)	MPEAMTIVML	0.30	NA	0/18	Yes[3]
NS3 (588-596)	APGGAKKPL	0.30	13.5	0/18	
NS3 (308-318)	RARANESATIL	0.30	3.7	0/18	
NS5 (209-219)	NPLSRNSTHEM	0.30	2.6	0/18	
NS4B (84-93)	IPFMKMNISV	0.30	NA	ND	
NS4B (84-94)	IPFMKMNISVI	0.30	NA	ND	
NS2A (6-13)	VPFGLVSM	0.30	0.4	0/16	
CapsidC (37-44)	RPGPSRGV	0.30	NA	ND	
EnvE (38-45)	KPSLDISL	0.40	NA	ND	
NS4A (73-80)	SPKGISRM	0.40	1.4	0/18	Yes[2]
NS1 (125-133)	SPGRKNGSF	0.40	2.7	0/18	
NS3 (227-236)	APTRVVLSEM	0.40	5.3	0/18	
NS1 (143-153)	CPFSNRVWNSF	0.40	1.0	0/16	
NS4B (51-58)	SPMLHHWI	0.40	NA	ND	
NS1 (243-252)	MPRSIGGPVS	0.40	NA	ND	
NS5 (583-591)	RPAPGGKAY	0.40	6.2	0/18	
CapsidC 37-47)	RPGPSRGVQGF	0.40	NA	ND	
NS2B (71-78)	SARYDVAL	0.40	NA	ND	
PrM (83-89))	RSRRSRRAI	0.40	NA	ND	
NS2B (92-102)	VPWDQVVMTSL	0.40	NA	ND	
NS4B (205-214)	GPLIEGNTSL	0.40	1.7	0/17	
EnvE ((52-60)	RPAEVRKVC	0.40	NA	ND	
NS5 672-681)	RPIDDRFGLA	0.80	3.1	8/46	Yes
EnvE (284-292)	RVKLSALTL	1.50	4.8	5/41	Yes

Table SIII. "hybrid forward-reverse immunology" vs "reverse immunology" approaches to HLA-B*07:02-restricted T cell epitope discovery.

NetMHCpan 2.4 was used to identify YFV-derived peptides predicted to bind with a high affinity to HLA-B*07:02 (threshold <0.5% Rank), and a selection of these were tested experimentally for their stability of HLA-B*07:02 binding, their ability to support peptide-HLA-B*07:02 tetramer generation, and their ability to stain CD8+ T cells obtained *ex vivo* from YFV vaccinees.

Note: HFRI suffix means that the peptides with the same suffix (1, 2 or 3) were found within the same 15mer peptide

Proteom position	Protein position	Sequence	Positive donors of 50 tested
YFV_pp9-23	Capsid C (9-23)	KTLGVNMVRRGVRSL	3
YFV_pp13-27	Capsid C (13-27)	VNMVRRGVRSLSNKI	9
YFV_pp17-31	Capsid C (17-31)	RRGVRSLSNKIKQKT	14
YFV_pp49-63	Capsid C (49-63)	FFFLFNILTGKKITA	11
YFV_pp53-67	Capsid C (53-67)	FNILTGKKITAHLKR	9
YFV_pp57-71	Capsid C (57-71)	TGKKITAHLKRLWKM	3
YFV_pp61-75	Capsid C (61-75)	ITAHLKRLWKMLDPR	4
YFV_pp65-79	Capsid C (65-79)	LKRLWKMLDPRQGLA	6
YFV_pp73-87	Capsid C (73-87)	DPRQGLAVLRKVKRV	4
YFV_pp77-91	Capsid C (77-91)	GLAVLRKVKRVVASL	4
YFV_pp81-95	Capsid C (81-95)	LRKVKRVVASLMRGL	10
YFV_pp85-99	Capsid C (85-99)	KRVVASLMRGLSSRK	2
YFV_pp121-135	prM (1-14)	GVTLVRKNRWLLLNV	1
YFV_pp125-139	prM (4-18)	VRKNRWLLLNVTSED	1
YFV_pp133-147	prM (12-26)	LNVTSEDLGKTFSVG	1
YFV_pp201-215	prM (80-94)	SAGRSRRSRRAIDLP	1
YFV_pp205-219	prM (84-98)	SRRSRRAIDLPTHEN	2
YFV_pp213-227	prM (92-106)	DLPTHENHGLKTRQE	1
YFV_pp217-231	prM (96-110)	HENHGLKTRQEKWMT	1
YFV_pp221-235	prM (100-114)	GLKTRQEKWMTGRMG	1
YFV_pp237-251	prM (116-130)	RQLQKIERWFVRNPF	2
YFV_pp245-259	prM (124-138)	WFVRNPFFAVTALTI	1
YFV_pp249-263	prM (128-142KK)	NPFFAVTALTIAYLVKK	3
YFV_pp253-267	prM (132-146K)	AVTALTIAYLVGSNMK	4
YFV_pp257-271	prM (136-150KK)	KKLTIAYLVGSNMTQRV	1
YFV_pp265-279	prM (144-158KK)	SNMTQRVVIALLVLAKK	1
YFV_pp285-299	EnvE (1-13)	SAHCIGITDRDFIEG	1
YFV_pp301-315	EnvE (16-30)	HGGTWVSATLEQDKC	2
YFV_pp329-343	EnvE (44-58)	SLETVAIDRPAEVRK	22
YFV_pp333-347	EnvE (48-62)	VAIDRPAEVRKVCYN	5
YFV_pp341-355	EnvE (56-70)	VRKVCYNAVLTHVKI	6
YFV_pp345-359	EnvE (60-74)	CYNAVLTHVKINDKC	4
YFV_pp409-423	EnvE (124-138)	SMSLFEVDQTKIQYV	4
YFV_pp413-427	EnvE (128-142)	FEVDQTKIQYVIRAQ	6
YFV_pp417-431	EnvE (132-146)	QTKIQYVIRAQLHVG	4
YFV_pp421-435	EnvE (136-150)	QYVIRAQLHVGAKQE	9
YFV_pp489-503	EnvE (204-218)	IVDRQWAQDLTLPWQ	2
YFV_pp493-507	EnvE (208-222)	QWAQDLTLPWQSGSG	1
YFV_pp505-519	EnvE (220-234)	GSGGVWREMHHLVEF	2
YFV_pp509-523	EnvE (224-238)	VWREMHHLVEFEPPH	3
YFV_pp525-539	EnvE (240-254)	ATIRVLALGNQEGSL	3
YFV_pp537-551	EnvE (252-266)	GSLKTALTGAMRVTK	1
YFV_pp553-567	EnvE (268-282)	TNDNNLYKLHGGHVS	1
YFV_pp557-571	EnvE (272-286)	NLYKLHGGHVSCRVK	9
YFV_pp573-587	EnvE (288-302)	SALTLKGTSYKICTD	2
YFV_pp589-603	EnvE (304-318)	MFFVKNPTDTGHGTV	4

YFV_pp601-615	EnvE (316-330)	GTVVMQVKVSKGAPC	4
YFV_pp613-627	EnvE (328-342)	APCRIPVIVADDLTA	1
YFV_pp625-639	EnvE (340-354)	LTAAINKGILVTVNP	3
YFV_pp629-643	EnvE (344-358)	INKGILVTVNPIAST	1
YFV_pp669-683	EnvE (384-398)	RLTYQWHKEGSSIGK	2
YFV_pp673-687	EnvE (388-402)	QWHKEGSSIGKLFTQ	6
YFV_pp681-695	EnvE (396-410)	IGKLFTQTMKGVERL	4
YFV_pp733-747	EnvE (KK448-462)	KKGLNWITKVIMGAVLI	1
YFV_pp745-759	EnvE (460-474K)	VLIWVGINTRNMTMSK	3
YFV_pp809-823	NS1 (31-45)	KYSYYPEDPVKLASI	3
YFV_pp841-855	NS1 (63-77)	LEHEMWRSRADEINA	3
YFV_pp845-859	NS1 (67-81)	MWRSRADEINAIFEE	4
YFV_pp849-863	NS1 (71-85)	RADEINAIFEENEVD	4
YFV_pp865-879	NS1 (87-101)	SVVVQDPKNVYQRGT	7
YFV_pp889-903	NS1 (111-125)	LQYGWKTWGKNLVFS	4
YFV_pp893-907	NS1 (115-129)	WKTWGKNLVFSPGRK	2
YFV_pp905-919	NS1 (127-140)	GRKNGSFIIDGKSRK	2
YFV_pp909-923	NS1 (131-144)	GSFIIDGKSRKECPF	4
YFV_pp921-935	NS1 (143-157)	CPFSNRVWNSFQIEE	1
YFV_pp945-959	NS1 (167-181)	VYMDAVFEYTIDCDG	2
YFV_pp953-967	NS1 (175-189)	YTIDCDGSILGAAVND	2
YFV_pp985-999	NS1 (207-221)	VNGTWMIHTLEALDY	5
YFV_pp989-1003	NS1 (211-225)	WMIHTLEALDYKECE	3
YFV_pp1001-1015	NS1 (223-237)	ECEWPLTHTIGTSVE	2
YFV_pp1005-1019	NS1 (227-241)	PLTHTIGTSVEESEM	1
YFV_pp1085-1099	NS1 (307-321)	VIPEWCCRSCTMPPV	1
YFV_pp1105-1119	NS1 (327-341)	DGCWYPMEIRPRKTH	4
YFV_pp1129-1143	NS2A (1-13)	TAGEIHAVPFGLVSM	1
YFV_pp1133-1147	NS2A (3-17KK)	IHAVPFGLVSMMIAMKK	1
YFV_pp1141-1155	NS2A (11-25)	VSMMIAMEVVLRKRQ	1
YFV_pp1177-1191	NS2A (47-61KK)	KKTLLDLLKLTVAVGLH	4
YFV_pp1181-1195	NS2A (51-65)	LLKLTVAVGLHFHEM	1
YFV_pp1197-1211	NS2A (KK67-80)	KKNGGDAMYMALIAAFS	1
YFV_pp1217-1231	NS2A (87-101)	LIGFGLRTLWSPRER	3
YFV_pp1229-1243	NS2A (99-113)	RERLVLTLGAAMVEI	1
YFV_pp1233-1247	NS2A (103-117KK)	VLTLGAAMVEIALGGKK	1
YFV_pp1245-1259	NS2A (115-129)	LGGVMGGLWKYLNAV	1
YFV_pp1249-1263	NS2A (119-133)	MGGLWKYLNAVSLCI	7
YFV_pp1253-1267	NS2A (123-137KK)	KKWKYLNAVSLCILTIN	4
YFV_pp1337-1351	NS2A (207-221)	QPFLGLCAFLATRIFK	1
YFV_pp1373-1387	NS2B (19-33)	GLAFQEMENFLGPIA	1
YFV_pp1377-1391	NS2B (23-37)	QEMENFLGPIAVGGL	1
YFV_pp1385-1399	NS2B (31-45KK)	KKPIAVGGLLMMLVSVA	1
YFV_pp1485-1499	NS3 (1-15)	SGDVLWDIPTPKIIE	2
YFV_pp1489-1503	NS3 (5-19)	LWDIPTPKIIEECEH	1
YFV_pp1505-1519	NS3 (21-35)	EDGIYGIFQSTFLGA	2
YFV_pp1529-1543	NS3 (45-59)	GGVFHTMWHVTRGAF	1
YFV_pp1533-1547	NS3 (49-63)	HTMWHVTRGAFLVRN	11
YFV_pp1537-1551	NS3 (53-67)	HVTRGAFLVRNGKKL	1

YFV_pp1541-1555	NS3 (57-71)	GAFLVRNGKKLIPSW	1
YFV_pp1581-1595	NS3 (97-111)	VQLIAAVPGKNVVNV	1
YFV_pp1629-1643	NS3 (145-159)	RNGEVIGLYGNGILV	2
YFV_pp1633-1647	NS3 (149-163)	VIGLYGNGILVGDNS	6
YFV_pp1641-1655	NS3 (157-171)	ILVGDNSFVSAISQT	1
YFV_pp1685-1699	NS3 (201-215)	GAGKTRRFLPQILAE	4
YFV_pp1689-1703	NS3 (205-219)	TRRFLPQILAECARR	5
YFV_pp1693-1707	NS3 (209-223)	LPQILAECARRRLRT	8
YFV_pp1697-1711	NS3 (213-227)	LAECARRRLRTLVLA	1
YFV_pp1701-1715	NS3 (217-231)	ARRRLRTLVLAPTRV	1
YFV_pp1713-1727	NS3 (229-243)	TRVVLSEMKEAFHGL	1
YFV_pp1737-1751	NS3 (253-267)	SAHGSGREVIDAMCH	1
YFV_pp1753-1767	NS3 (269-283)	TLTYRMLEPTRVVNW	4
YFV_pp1765-1779	NS3 (281-295)	VNWEVIIMDEAHFLD	15
YFV_pp1769-1783	NS3 (285-299)	VIIMDEAHFLDPASI	10
YFV_pp1773-1787	NS3 (289-303)	DEAHFLDPASIAARG	1
YFV_pp1777-1791	NS3 (293-307)	FLDPASIAARGWAAH	1
YFV_pp1797-1811	NS3 (313-327)	ESATILMTATPPGTS	1
YFV_pp1821-1835	NS3 (337-351)	IEDVQTDIPSEPWNT	3
YFV_pp1833-1847	NS3 (349-363)	WNTGHDWILADKRPT	3
YFV_pp1837-1851	NS3 (353-367)	HDWILADKRPTAWFL	16
YFV_pp1841-1855	NS3 (357-371)	LADKRPTAWFLPSIR	2
YFV_pp1845-1859	NS3 (361-375)	RPTAWFLPSIRAANV	2
YFV_pp1849-1863	NS3 (365-379)	WFLPSIRAANVMAAS	10
YFV_pp1853-1867	NS3 (369-383)	SIRAANVMAASLRKA	8
YFV_pp1861-1875	NS3 (377-391)	AASLRKAGKSVVVLNK	1
YFV_pp1869-1883	NS3 (385-399)	KSVVVLNRKTFEREY	4
YFV_pp1877-1891	NS3 (393-407)	KTFEREYPTIKQKKP	4
YFV_pp1897-1911	NS3 (413-427)	TDIAEMGANLCVERV	1
YFV_pp1929-1943	NS3 (445-459)	VAIKGPLRISASSAA	1
YFV_pp1933-1947	NS3 (449-463)	GPLRISASSAAQRRG	3
YFV_pp1941-1955	NS3 (457-471)	SAAQRRGRIGRNPNR	2
YFV_pp1945-1959	NS3 (461-475)	RRGRIGRNPNRDGDS	2
YFV_pp1957-1971	NS3 (473-487)	GDSYYYSEPTSENNA	4
YFV_pp1961-1975	NS3 (477-491)	YYSEPTSENNAHHVC	2
YFV_pp1969-1983	NS3 (485-499)	NNAHHVCWLEASMLL	2
YFV_pp1973-1987	NS3 (489-503)	HVCWLEASMLLDNME	1
YFV_pp1989-2003	NS3 (505-519)	RGGMVAPLYGVEGTK	1
YFV_pp2021-2035	NS3 (537-551)	FRELVRNCDLPVWLS	1
YFV_pp2029-2043	NS3 (545-559)	DLPVWLSWQVAKAGL	2
YFV_pp2033-2047	NS3 (549-563)	WLSWQVAKAGLKTND	1
YFV_pp2185-2199	NS4A (78-92)	SRMSMAMGTMAGCGY	1
YFV_pp2189-2203	NS4A (82-96K)	MAMGTMAGCGYLMFLK	2
YFV_pp2285-2299	NS4B (29-43)	WPDLDLKPGAAWTVY	1
YFV_pp2289-2303	NS4B (33-47)	DLKPGAAWTVYVGIV	3
YFV_pp2293-2307	NS4B (37-51K)	GAAWTVYVGIVTMLSK	1
YFV_pp2297-2311	NS4B (41-55K)	TVYVGIVTMLSPMLHK	5
YFV_pp2329-2343	NS4B (73-87)	SASVLSFMDKGIPFM	1
YFV_pp2337-2351	NS4B (81-95K)	DKGIPFMKMNISVIMK	4

YFV_pp2341-2355	NS4B (85-99KK)	KKPFMKMNISVIMLLVS	1
YFV_pp2353-2367	NS4B (97-111)	LVSGWNSITVMPLLC	4
YFV_pp2357-2371	NS4B (101-115KK)	KKWNSITVMPLLCGIGC	1
YFV_pp2381-2395	NS4B (125-139)	PGIKAQQSKLAQRRV	2
YFV_pp2385-2399	NS4B (129-143)	AQQSKLAQRRVFHGV	1
YFV_pp2389-2403	NS4B (133-147 (E->K)	KLAQRRVFHGVAKNP	5
YFV_pp2393-2407	NS4B (137-151 (E->K)	RRVFHGVAKNPVVDG	4
YFV_pp2397-2411	NS4B (141-155 (E->K)	HGVAKNPVVDGNPTV	1
YFV_pp2401-2415	NS4B (146-159)	KNPVVDGNPTVDIEE	2
YFV_pp2405-2419	NS4B (149-163)	VDGNPTVDIEEAPEM	1
YFV_pp2425-2439	NS4B (KK169-183)	KKKKLALYLLLALSLAS	1
YFV_pp2437-2451	NS4B (181-195)	LASVAMCRTPFSLAE	6
YFV_pp2481-2495	NS4B (225-239)	TGVMRGNHYAFVGVM	1
YFV_pp2485-2499	NS4B (229-241)	RGNHYAFVGVMYNLW	1
YFV_pp2489-2503	NS4B (233-247K)	YAFVGVMYNLWKMKTK	10
YFV_pp2513-2527	NS5 (7-21)	TLGEVWKRELNLLDK	2
YFV_pp2517-2531	NS5 (11-25)	VWKRELNLLDKRQFE	1
YFV_pp2529-2543	NS5 (23-37)	QFELYKRTDIVEVDR	4
YFV_pp2565-2579	NS5 (59-73)	TAKLRWFHERGYVKL	2
YFV_pp2569-2583	NS5 (63-77)	RWFHERGYVKLEGRV	1
YFV_pp2573-2587	NS5 (67-81)	ERGYVKLEGRVIDLG	2
YFV_pp2677-2691	NS5 (171-185K)	WLACGVDNFCVKVLAK	3
YFV_pp2833-2847	NS5 (327-341)	KILTYPWDRIEEVTR	1
YFV_pp2877-2891	NS5 (371-385)	TRKIMKVVNRWLFRH	11
YFV_pp2881-2895	NS5 (375-389)	MKVVNRWLFRHLARE	3
YFV_pp2885-2899	NS5 (379-393)	NRWLFRHLAREKNPR	3
YFV_pp2905-2919	NS5 (399-413)	EFIAKVRSHAAIGAY	1
YFV_pp2957-2971	NS5 (451-465)	CVYNMMGKREKKLSE	1
YFV_pp2977-2991	NS5 (471-485)	GSRAIWYMWLGARYL	10
YFV_pp2981-2995	NS5 (475-489(KK)	IWYMWLGARYLEFEAKK	8
YFV_pp2989-3003	NS5 (483-497)	RYLEFEALGFLNEDH	1
YFV_pp3057-3071	NS5 (551-565)	EQEILNYMSPHHKKL	5
YFV_pp3061-3075	NS5 (555-569)	LNYMSPHHKKLAQAV	2
YFV_pp3069-3083	NS5 (563-577)	KKLAQAVMEMTYKNK	1
YFV_pp3073-3087	NS5 (567-581)	QAVMEMTYKNKVVKV	2
YFV_pp3077-3091	NS5 (571-585)	EMTYKNKVVKVLRPA	2
YFV_pp3117-3131	NS5 (611-625)	LNTITNLKVQLIRMA	3
YFV_pp3145-3159	NS5 (639-653(KK)	KKCDESVLTRLEAWLTE	1
YFV_pp3205-3219	NS5 (699-713)	QPSKGWNDWENVPFC	1
YFV_pp3213-3227	NS5 (707-727)	WENVPFCSHHFHELQ	1
YFV_pp3277-3291	NS5 (771-785)	FHKRDMRLLSLAVSS	3
YFV_pp3281-3295	NS5 (775-789)	DMRLLSLAVSSAVPT	4
YFV_pp3313-3327	NS5 (807-821)	WMTTEDMLEVWNRVW	1
YFV_pp3369-3383	NS5 (863-877)	WASHIHLVIHRIRTL	2
YFV_pp3373-3387	NS5 (867-881)	IHLVIHRIRTLIGQE	13
YFV_pp3377-3391	NS5 (871-885)	IHRIRTLIGQEKYTD	9

Supplementary Table IV: List of 192 $CD4^+$ T cell stimulatory 15mer epitopes

Proteome position	Protein position	Sequence	TMR validated HLA-II restriction	Predicted affinity (%RANK)	Measured affinity (nM)	Responders /donors tested
YFV_pp17-31	Capsid C (17-31)	RRGVRSLSNKIKQKT	DRB5*01:01	0.50	4	6/6
YFV_pp49-63	Capsid C (49-63)	FFFLFNILTGKKITA	DRB1*01:01	0.70	27	8/8
YFV_pp53-67	Capsid C (53-67)	FNILTGKKITAHLKR	DRB1*01:01	6.50	7	8/8
YFV_pp65-79	Capsid C (65-79)	LKRLWKMLDPRQGLA	DRB1*01:01	4.50	15	5/5
YFV_pp73-87	Capsid C (73-87)	DPRQGLAVLRKVKRV	DRB1*13:01	0.12	4	1/1
YFV_pp73-87	Capsid C (73-87)	DPRQGLAVLRKVKRV	DRB1*13:02	32.00	5	5/5
YFV_pp77-91	Capsid C (77-91)	GLAVLRKVKRVVASL	DRB1*14:54	0.17	6	1/1
YFV_pp81-95	Capsid C (81-95)	LRKVKRVVASLMRGL	DRB1*07:01	0.70	7	5/5
YFV_pp81-95	Capsid C (81-95)	LRKVKRVVASLMRGL	DRB1*11:01	0.25	4	2/2
YFV_pp85-99	Capsid C (85-99)	KRVVASLMRGLSSRK	DRB1*11:01	0.12	5	1/1
YFV_pp237-251	prM (116-130)	RQLQKIERWFVRNPF	DRB1*14:54	3.50	8	1/1
YFV_pp329-343	EnvE (44-58)	SLETVAIDRPAEVRK	DRB1*03:01	0.40	21	14/15
YFV_pp329-343	EnvE (44-58)	SLETVAIDRPAEVRK	DRB3*03:01	9.00	21	11/11
YFV_pp333-347	EnvE (48-62)	VAIDRPAEVRKVCYN	DRB3*03:01	32.00	12	5/7
YFV_pp333-347	EnvE (48-62)	VAIDRPAEVRKVCYN	DRB1*03:01	11.00	15	4/5
YFV_pp341-355	EnvE (56-70)	VRKVCYNAVLTHVKI	DRB5*01:01	1.30	4	6/6
YFV_pp345-359	EnvE (60-74)	CYNAVLTHVKINDKC	DRB5*01:01	7.00	9	5/5
YFV_pp417-431	EnvE (132-146)	QTKIQYVIRAQLHVG	DRB1*14:54	1.90	23	1/1
YFV_pp525-539	EnvE (240-254)	ATIRVLALGNQEGSL	DRB1*04:04	4.50	22	2/2
YFV_pp557-571	EnvE (272-286)	NLYKLHGGHVSCRVK	DRB1*13:02	44.00	9	7/7
YFV_pp673-687	EnvE (288-402)	QWHKEGSSIGKLFTQ	DRB1*04:01	43.00	567	2/2
YFV_pp601-615	EnvE (316-330)	GTVVMQVKVSKGAPC	DRB1*04:04	13.00	16	1/1
YFV_pp625-639	EnvE (340-354)	LTAAINKGILVTVNP	DRB3*03:01	1.50	2	5/5
YFV_pp865-879	NS1 (87-101)	SVVVQDPKNVYQRGT	DRB1*03:01	0.80	10	5/5
YFV_pp889-903	NS1 (111-125)	LQYGWKTWGKNLVFS	DRB1*13:01	6.50	6	1/1
YFV_pp889-903	NS1 (111-125)	LQYGWKTWGKNLVFS	DRB1*13:02	30.00	9	4/4
YFV_pp893-907	NS1 (115-129)	WKTWGKNLVFSPGRK	DRB1*13:02	34.00	5	3/3
YFV_pp1105-1119	NS1 (327-341)	DGCWYPMEIRPRKTH	DRB5*01:01	1.50	2	8/8
YFV_pp1373-1387	NS2B (19-33)	GLAFQEMENFLGPIA	DRB1*15:01	16.00	42	1/1
YFV_pp1533-1547	NS3 (49-63)	HTMWHVTRGAFLVRN	DRB1*07:01	0.01	3	8/8
YFV_pp1541-1555	NS3 (57-71)	GAFLVRNGKKLIPSW	DRB1*13:01	0.06	2	1/1
YFV_pp1541-1555	NS3 (57-71)	GAFLVRNGKKLIPSW	DRB1*13:02	1.70	3	1/1
YFV_pp1541-1555	NS3 (57-71)	GAFLVRNGKKLIPSW	DRB1*14:54	2.50	6	1/1
YFV_pp1633-1647	NS3 (149-163)	VIGLYGNGILVGDNS	DRB1*15:01	18.00	14	4/4
YFV_pp1765-1779	NS3 (281-295)	VNWEVIIMDEAHFLD	DRB1*03:01	0.07	43	5/5
YFV_pp1769-1783	NS3 (285-299)	VIIMDEAHFLDPASI	DRB1*03:01	0.10	13	6/6
YFV_pp1765-1779	NS3 (281-295)	VNWEVIIMDEAHFLD	DRB3*01:01	0.02	3	8/10
YFV_pp1769-1783	NS3 (285-299)	VIIMDEAHFLDPASI	DRB3*01:01	0.08	8	1/1
YFV_pp1765-1779	NS3 (281-295)	VNWEVIIMDEAHFLD	DRB3*03:01	4.00	15	4/4
YFV_pp1769-1783	NS3 (285-299)	VIIMDEAHFLDPASI	DRB3*03:01	6.00	11	4/4
YFV_pp1833-1847	NS3 (349-363)	WNTGHDWILADKRPT	DRB1*03:01	8.50	28	3/3
YFV_pp1837-1851	NS3 (353-367)	HDWILADKRPTAWFL	DRB1*03:01	0.01	2	12/12
YFV_pp1849-1863	NS3 (365-379)	WFLPSIRAANVMAAS	DRB1*04:04	0.08	4	1/1
YFV_pp1853-1867	NS3 (369-383)	SIRAANVMAASLRKA	DRB1*04:04	0.80	13	1/1
YFV_pp1877-1891	NS3 (393-407)	KTFEREYPTIKQKKP	DRB5*01:01	11.00	2	5/5
YFV_pp2021-2035	NS3 (537-551)	FRELVRNCDLPVWLS	DRB3*03:01	0.70	5	1/2
YFV_pp2337-2351	NS4B (81-95K)	DKGIPFMKMNISVIMK	DRB3*03:01	0.15	3	5/5
YFV_pp2401-2415	NS4B (146-159)	KNPVVDGNPTVDIEE	DRB3*03:01	44.00	7	5/5
YFV_pp2405-2419	NS4B (149-163)	VDGNPTVDIEEAPEM	DRB3*03:01	90.00	22	5/5
YFV_pp2437-2451	NS4B (181-195)	LASVAMCRTPFSLAE	DRB1*04:04	0.04	2	1/1
YFV_pp2489-2503	NS4B (233-247K)	YAFVGVMYNLWKMKTK	DPA1*01:03-DPB1*04:01	5.00	NA	4/4
YFV_pp2565-2579	NS5 (59-73)	TAKLRWFHERGYVKL	DRB1*13:01	12.00	7	1/1
YFV_pp2565-2579	NS5 (59-73)	TAKLRWFHERGYVKL	DRB1*13:02	47.00	10	1/2
YFV_pp2565-2579	NS5 (59-73)	TAKLRWFHERGYVKL	DRB5*01:01	3.00	4	4/4
YFV_pp2569-2583	NS5 (63-77)	RWFHERGYVKLEGRV	DRB5*01:01	5.50	5	1/1
YFV_pp2877-2891	NS5 (371-385)	TRKIMKVVNRWLFRH	DRB1*15:01	0.60	7	5/5
YFV_pp2905-2919	NS5 (399-413)	EFIAKVRSHAAIGAY	DRB1*15:01	1.20	2	1/1
YFV_pp2977-2991	NS5 (471-485)	GSRAIWYMWLGARYL	DRB1*01:01	6.50	6	8/8
YFV_pp2981-2995	NS5 (475-489KK)	IWYMWLGARYLEFEAKK	DRB1*01:01	17.00	4	8/8
YFV_pp3057-3071	NS5 (551-565)	EQEILNYMSPHHKKL	DRB1*15:01	0.15	29	5/5
YFV_pp3057-3071	NS5 (551-565)	EQEILNYMSPHHKKL	DRB5*01:01	0.08	2	5/5
YFV_pp3061-3075	NS5 (555-569)	LNYMSPHHKKLAQAV	DRB5*01:01	0.50	2	3/3
YFV_pp3373-3387	NS5 (867-881)	IHLVIHRIRTLIGQE	DRB1*04:04	0.10	14	2/2
YFV_pp3377-3391	NS5 (871-885)	IHRIRTLIGQEKYTD	DRB1*04:04	1.80	27	2/2

Table SV. A complete list of the 50 CD4$^+$ T cell epitopes identified by the HFRI approach.

The CD4$^+$ T cell epitope discovery process detailed for donor YF1067 was extended to 50 randomely selected, primary YFV vaccinated donors. 50 different CD4$^+$ T cell responses restricted by 14 HLA-II allotypes (13 HLA-DR-restricted and one HLA-DP-restricted) were discovered. The peptide-HLA-I affinities were predicted by NetMHCIIpan (given in %Rank) and the binding affinity was measured (given in nM) (the lower the prediction or measurement, the better the affinity). Productively interacting peptide-HLA-II combinations were used to design and generate peptide-HLA class II tetramers. The resulting tetramers were used to stain and analyze expanded CD4+ T cells by flow cytometry gating on CD3+ CD4+ T cells. The prevalence of these responses is given as "the number of responding donors/the number of donors tested".

In the event that two overlapping peptides were identified, the overlapping sequence has been colored red. For solubilization purpose, lysines have in some cases been added to the N- or C-terminal of the peptide (indicated here by underlining these added lysines.
